# An Evaluation of Passive and Active Approaches to Improve Tuberculosis Notifications in Afghanistan

**DOI:** 10.1371/journal.pone.0163813

**Published:** 2016-10-04

**Authors:** A. Sanaie, C. Mergenthaler, A. Nasrat, M. K. Seddiq, S. D. Mahmoodi, R. H. Stevens, J. Creswell

**Affiliations:** 1 Anti-TB Association Afghanistan, Kabul, Afghanistan; 2 Stop TB Partnership, Geneva, Switzerland; 3 ACREOD, Kabul, Afghanistan; 4 National TB Program, Kabul, Afghanistan; 5 Mott McDonald, London, United Kingdom; University of Cape Town, SOUTH AFRICA

## Abstract

**Background:**

In Afghanistan, improving TB case detection remains challenging. In 2014, only half of the estimated incident TB cases were notified, and notifications have decreased since peaking in 2007. Active case finding has been increasingly considered to improve TB case notifications. While access to health services has improved in Afghanistan, it remains poor and many people seeking health services won’t receive proper care.

**Methods:**

From October 2011 through December 2012 we conducted three separate case finding strategies in six provinces of Afghanistan and measured impact on TB case notification. Systematically screening cough among attendees at 47 health facilities, active household contact investigation of smear-positive index TB patients, and active screening at 15 camps for internally displaced people were conducted. We collected both intervention yield and official quarterly notification data. Additional TB notifications were calculated by comparing numbers of cases notified during the intervention with those notified before the intervention, then adjusting for secular trends in notification.

**Results:**

We screened 2,022,127 people for TB symptoms during the intervention, tested 59,838 with smear microscopy and detected 5,046 people with smear-positive TB. Most cases (81.7%, 4,125) were identified in health facilities while nearly 20% were found through active case finding. A 56% increase in smear-positive TB notifications was observed between the baseline and intervention periods among the 47 health facilities, where cases detected by all three strategies were notified.

**Discussion:**

While most people with TB are likely to be identified through health facility screening, there are many people who remain without a proper diagnosis if outreach is not attempted. This is especially true in places like Afghanistan where access to general services is poor. Targeted active case finding can improve the number of people who are detected and treated for TB and can push towards the targets of the Stop TB Global Plan and End TB Strategy.

## Introduction

Afghanistan is ranked among the 30 countries with the highest tuberculosis (TB) burdens in the world. An estimated 60,000 Afghans developed TB and 14,000 died from the disease in 2014 [1]. During the past decade and a half, war has destroyed the country’s health infrastructure, and continued insecurity and political instability have hampered efforts to restore basic health services, including TB control [2]. Other factors putting Afghans at risk of becoming infected with and developing active TB include chronic poverty and geographical impediments that hinder access to basic health care for rural populations [3].

In spite of these challenges, in 2005 the National Tuberculosis Control Program (NTP) launched an operational plan to expand DOTS coverage into all comprehensive health centers (so named as they contained laboratories and medical doctors, and provided basic care services indicated by most disease programs), provincial hospitals in eight high-prevalence regions, and some basic health centers [4]. Community DOTS programs in comprehensive health centers were also planned and involved community health workers in TB control efforts, i.e. screening for symptoms, making house-to-house screening visits, and providing treatment support [5]. TB case notifications rose until 2007, and treatment outcomes improved from less than 50% treatment success in the late 1990’s to approximately 90% treatment success due to sustained efforts to strengthen TB control [2]. Despite progress in these areas, improving TB case detection has remained a major challenge in Afghanistan. Although the treatment success rate was 89% in 2010, only half of the estimated incident TB cases were notified to the NTP, and notifications have decreased since reaching a peak in 2007 [1].

Recently, much attention has been focused on the role of screening to improve TB case detection [6–8]. Programmatic interventions for improving case detection need to thoroughly assess the epidemiological context, identify and prioritise specific populations [7]. Improving TB case detection can be achieved by targeting identified breakdowns in the TB care pathway, and understanding which interventions work best to respond to those issues [9]. Two potential scenarios by which health systems can fail to identify people who are sick with TB are 1) when access to care is poor, especially in marginalized communities, and 2) when health care workers (HCW) fail to identify those with TB symptoms for diagnostic testing among those accessing care. ACF approaches that improve access to TB care [8,10–11] and better systematic screening inside health facilities [12–13] can contribute to improving case detection.

Although health care workers are expected to carry out diagnostic activities, their motivation and ability to perform these duties are often low because of poor compensation, and lack of training and support [14]. Similarly, most health providers in Afghanistan are poorly compensated and have little training, resources and experience needed for TB diagnosis and treatment [5]. In 2013 Afghanistan had at least an estimated 631,000 internally displaced people (IDP), comprising more than 2% of the population and one of the highest numbers in the world [15]. Many IDPs live in poor conditions in camps and lack proper access to even basic health facilities [3]. Although access to health care has improved in the country, it remains insufficient as more than 40% of the population lives more than an hour away from any health facility [16].

Given the low case detection rate and poor access to health services in Afghanistan, we conducted a programmatic intervention to improve TB case detection through three separate case finding approaches. Our aims were to evaluate the impact of different active and passive case finding strategies, on both strategy-specific case finding yield and on provincial level TB case notifications.

## Methods

### Interventions

The intervention was based in six Afghan provinces: Nangarhar, Laghman, Kunar, Kandahar, Paktia and Faryab, with a total estimated population of 4.5 million and a total of 124 health facilities providing TB services in 2011. Three separate case finding approaches were used: systematic screening among attendees of 47 of the 124 BMU health facilities, active household contact investigation of SS+ TB patients from the 47 facilities, and ACF through outreach and screening at 15 IDP camps. These 47 facilities were selected as they were already supported by the NTP and the implementing NGO, were run by dedicated staff in the context of a struggling health system, yet had very low TB case notification rates. Cases detected among those living in the IDP camps were notified in one of the 47 health facilities. Case finding activities were conducted from October 2010 through December 2012; therefore, “year one” and “year two” refer to four and five annual quarters of case finding activities, respectively.

### Screening and testing algorithms

Identifying people with suspected TB was done using the standard NTP approach. This means that people were asked about presence of cough of two weeks or more and those responding affirmatively were eligible for diagnostic testing with sputum smear microscopy (SSM) using light microscopes. Health facility screening was done systematically to all attendees of the 47 facilities. People identified as needing diagnostic testing for TB were requested to provide three sputum samples, and those with at least two positive samples were eligible for anti-TB treatment. Samples were collected according to NTP guidelines: spot, morning then spot. Individuals with one positive sample were referred for chest x-ray (CXR) and follow-up.

Because of costs and logistics for active outreach to IDPs and household contacts, a total of two sputum samples were collected and transported to the nearest laboratory for testing. One spot followed by a morning sample were collected from IDPs, while two spot samples were collected from household contacts. For the IDP screening a mobile team first coordinated with the camp chiefs and mapped out the camps to ensure no homes were missed, carried out door-to-door visits, screening all members present in the dwelling. To treat SS+ individuals identified among IDPs, TB medicines were delivered to the camps, and volunteer treatment supporters oversaw treatment. Two outreach workers also conducted home visits for contact investigation of people identified with SS+ TB at one of the 47 facilities. A household contact was defined as any person living in the same home as the SS+ index case at the time of his/her visit to the health facility.

Individuals identified with TB were registered for treatment and monitored at the 47 health facilities while 75 trained volunteer TB patient treatment supporters provided directly observed treatment across the 15 IDP camps.

### Data Analysis

De-identified data were collected through standardized reporting registers that tracked NTP case notification data over three pre-intervention years comprising the historical baseline; this continued throughout the intervention period. The case finding yield of the intervention was disaggregated by the three approaches. The number needed to screen (NNS) i.e. the number of people that had to be screened for symptoms to identify one person with TB, was calculated for each of the three case-finding activities. For year on year changes among individual case finding interventions, we tested for associations of significance using Pearson chi-square two-tailed tests. To predict the expected case notifications in the absence of the intervention, linear regression was used to project future notifications based on the three-year historical baseline period. Additional TB notifications were calculated by comparing the number of cases notified to the NTP during the intervention period with those notified during the historical baseline, and adjusted for secular trends in notifications. All analyses were done using Stata version 12.1.

Prior to analysis, this intervention only used de-identified (anonymized) data from registers that were collected as part of routine NTP practice. We obtained administrative approval from each of the intervention area’s six provincial health directorates (Nangarhar, Laghman, Kunar, Kandahar, Paktia and Faryab) and from Afghanistan’s NTP (National Tuberculosis Control Program), but did not require any additional ethical clearance as no additional patient level data was collected and reported outside NTP programmatic activities.

## Results

During the study period we screened 2,022,127 people for TB symptoms across all three interventions, 1,699,277 (84%) of whom were screened at health facilities (Table 1). Screening activities conducted through all interventions identified a total of 59,838 (3.0%) people who were tested with SSM. Among tested individuals, 46,763 (78.1%) were health facility attendees, 8,836 (14.8%) were IDPs, and 4,239 (7.1%) were household contacts. The proportion of screened individuals who were tested with SSM significantly increased among HHC and HF attendees, while significantly decreasing among IDPs between years 1 and 2 (p< 0.001 for all strategies). The proportion of SS+ results among tested individuals was 8.8% at health facilities, the highest proportion of the three interventions (Table 1). The proportional yield of SS+ cases identified only changed significantly among HHCs, decreasing from 9.19% in year 1 to 4.78% in year 2 (p<0.001). In the first year of implementation, we identified 2,480 people with SS+ TB across all strategies, while in the second year 2,566 people were identified. This proportional increase of SS+ cases among all screened individuals showed a slight increase from year one to year two (0.24% to 0.26%; p<0.001). Treatment outcomes were high, with treatment success at approximately 90%, and similar across both years of the intervention (S2 Dataset).

**Table 1:** Results of Tuberculosis Screening, Afghanistan 2010–2012.

	Year 1		Year 2			Total	
**Internally Displaced People**	N	(%)	N	(%)	P-value	N	(%)
Total Screened	155,897		150,308			306,205	15.14%
People tested with SSM (%)	5,139	3.30%	3,697	2.46%	**<0.001**	8,836	2.89%
Total Smear Positive	358	0.23%	295	0.20%	**0.045**	653	0.2%
Yield	6.97%		7.98%		0.073	7.39%	
NNS SS+	436		510			**469**	
Total All Forms TB	389		346		**0.003**	735	
NNS All Forms TB	401		434			**417**	
**Household Contacts**	N	(%)	N	(%)	P-value	N	(%)
Total Screened	7,232		9,413			16,645	0.82%
People tested with SSM	1,480	20.46%	2,759	29.31%	**<0.001**	4,239	25.47%
Total Smear Positive	136	1.88%	132	1.40%	**0.015**	268	1.6%
Yield	9.19%		4.78%		**<0.001**	6.32%	
NNS SS+	53		71			**62**	
Total All Forms TB	158		146		**<0.001**	304	
NNS All Forms TB	46		65			**55**	
**Health Facilities**	N	(%)	N	(%)	P-value	N	(%)
Total Screened	889,120		810,157			1,699,277	84.03%
People tested with SSM	22,228	2.50%	24,535	3.03%	**<0.001**	46,763	2.75%
Total Smear Positive	1,986	0.22%	2,139	0.26%	**<0.001**	4,125	0.2%
Yield	8.93%		8.72%		0.410	8.82%	
NNS SS+	448		379			**412**	
Total All Forms TB	3,540		4,584		**<0.001**	8,124	
NNS All Forms TB	251		177			**209**	
**All Strategies**	N	(%)	N	(%)	P-value	N	(%)
Total Screened	1,052,249		969,878			2,022,127	
People tested with SSM	28,847	2.74%	30,991	3.20%	**<0.001**	59,838	2.96%
Total Smear Positive	2,480	0.24%	2,566	0.26%	**<0.001**	5,046	0.25%
Yield	8.60%		8.28%		0.163	8.43%	
NNS SS+	424		378			**401**	
Total All Forms TB	4,087		5,076		**<0.001**	9,163	
NNS All Forms	257		191			**221**	

TB = Tuberculosis; NNS = Number needed to screen; SSM = Sputum smear microscopy; SS+ = Sputum smear-positive

### NNS and Gender

The NNS decreased for all strategies from 424 to 378 between years 1 and 2, as it did also for the major case finding approach in health facilities from 448 to 379. NNS increased among IDPs and HHCs, from 436 to 510 and 53 to 71, respectively. The lowest NNS was found among household contacts of TB patients. To identify one case of SS+ TB, 62 household contacts had to be screened (1.6% prevalence among contacts screened). In contrast, health facility screening required 412 people to detect one SS+ TB case (0.2% prevalence among screened health facility attendees), while IDP screening yielded one SS+ TB case for every 469 people screened (also 0.2% prevalence among screened IDPs) (Table 1).

Although similar proportions of males and females were screened among contacts and IDPs, the NNS for both strategies was lower among women (49 among contacts and 166 among IDPs) than men (86 among contacts and 272 among IDPs) (Tables 2 and 3). While NNS increased for both genders for both strategies from year 1 to year 2, the proportional SS+ yield did not increase significantly for either gender or strategy between years 1 and 2 (Tables 2 and 3). NNS could not be disaggregated by gender for screening in health facilities. Only the proportion screened and suspects tested through contact investigation increased significantly for men between years 1 and 2 (Table 2). In health facilities, the female to male (F/M) ratio for SS+ cases was 2.0 and 1.9 for years one and two, respectively. In IDP camps, the F/M ratio was 1.7 both years, while in household contact screening, the F/M ratio was 2.1 and 1.6 respectively. For detailed age and sex disaggregated intervention data, see S1 Dataset.

**Table 2:** Contact Investigation Screening By Gender, Afghanistan 2010–2012.

		Year 1	%	Year 2	%	P-value	Total	%
**Number Screened**	Men	3,471	48.0%	4,697	49.9%		8,168	49.1%
	Women	3,761	52.0%	4,716	50.1%		8,477	50.9%
	**Total**	**7,232**	** **	**9,413**	** **	**0.015**	**16,645**	
**People with suspected TB tested**	Men	666	45.0%	1,335	48.4%		2,001	47.2%
	Women	814	55.0%	1,424	51.6%		2,238	52.8%
	**Total**	**1,480**	** **	**2,759**	** **	**0.035**	**4,239**	
**SS+ Detected**	Men	44	32.4%	51	38.6%		95	35.4%
	Women	92	67.6%	81	61.4%		173	64.6%
	**Total**	**136**	** **	**132**	** **	0.282	**268**	
**NNS**	Men	79		92			86	
	Women	41		58			49	
	**Total**	**53**		**71**			**62**	

**Table 3:** Internally Displaced People Screening by Gender, Afghanistan 2010–2012.

		Year 1	%	Year 2	%	P-value	Total	%
**Number Screened**	Men	33,829	21.7%	32,617	21.7%		66,446	21.7%
	Women	34,590	22.2%	33,350	22.2%		67,940	22.2%
	**Total**	155,897*	** **	150,308*	** **	1.000	**306,205**	
**People with suspected TB tested**	Men	2,043	39.8%	1,468	39.7%		3,511	39.7%
	Women	3,096	60.2%	2,229	60.3%		5,325	60.3%
	**Total**	**5,139**	** **	**3,697**	** **	0.965	**8836**	
**SS+ Detected**	Men	134	37.4%	110	37.3%		244	37.4%
	Women	224	62.6%	185	62.7%		409	62.6%
	**Total**	**358**	** **	**295**	** **	0.970	**653**	
**NNS**	Men	253		297			272	
	Women	154		180			166	
	**Total**	**436**		**510**			**469**	

* Includes screened children

TB = Tuberculosis; SS+ = Sputum smear positive; NNS = Number needed to screen to find one case of SS+ TB (Screened/Detected)

### Impact on Case Notification

Notifications of SS+ TB in the intervention’s 47 health facilities increased 56% between the baseline and intervention periods. In the nine quarters prior to the interventions (also nine quarters) there were 3,097 SS+ TB cases notified while during the study 4,842 SS+ cases were notified. In all 124 facilities in the six provinces, there was a 17.4% increase in case notifications (5,876 to 6,901) between the baseline and intervention periods (Table 4).

**Table 4:** Tuberculosis Notifications in Afghanistan 2008–2012 and Impact of Case Finding Interventions.

			Baseline Period									Intervention Period								
	*2008*				*2009*				*2010*				*2011*				*2012*			
	*Q1*	*Q2*	*Q3*	*Q4*	*Q1*	*Q2*	*Q3*	*Q4*	*Q1*	*Q2*	*Q3*	*Q4*	*Q1*	*Q2*	*Q3*	*Q4*	*Q1*	*Q2*	*Q3*	*Q4*
**47 BMUs Total SS+ Cases**	397	425	356	309	409	347	298	324	323	378	353	**390**	**680**	**717**	**595**	**486**	**507**	**538**	**475**	**454**
			3,097									**4,842 (+56.3%)**								
**124 BMUs Total SS+ Cases**	648	704	582	514	705	695	582	642	746	767	643	**707**	**925**	**965**	**744**	**714**	**727**	**787**	**686**	**646**
** **			5,876									**6,901 (+17.4%)**								

BMU = Basic management unit; SS+ = Sputum smear-positive

**Fig 1:**
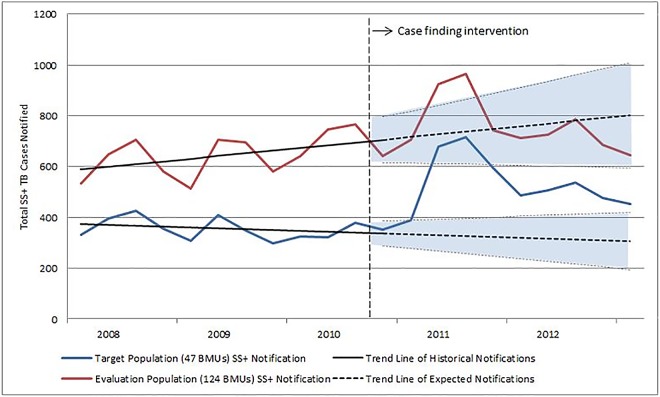
Smear Positive Tuberculosis Case Notifications in Afghanistan in 6 Provinces 2008–2012.

Projecting the declining secular trend of notifications among the 47 facilities in the baseline period forward through 2012, only 59% (2,885; 95% CI: 2,129–3,640) of the cases notified during the intervention period are accounted for. In contrast, doing the same exercise for the 124 BMUs of the evaluation population, the projected notifications account for 98% (6,829; 95% CI: 5,442–8,215) of the cases notified during the intervention period. Fig 1 demonstrates the negative trend in the historical case notification that was reversed during the intervention in the 47 facilities but a positive trend at the level of the six provinces. Fig 1 also shows that after an increase during 2011, notifications returned to an expected number based on the trend among the 124 facilities while in the 47 intervention facilities notifications continued to be higher than expected. Detailed case notification data from the 47 facilities can be found in S2 Dataset.

## Discussion

The results of our study highlight the large number of undiagnosed prevalent SS+ TB cases that can be reached and notified with a multifaceted package of case finding activities. The intervention’s effect was documented at the facility and provincial level, a finding which was strengthened by the linear projection’s indication that continued routine activities would have detected only 59% of cases notified during the intervention period in the target population. Other recent initiatives have also shown that intensified efforts to improve TB case detection can increase notifications across a wide variety of approaches and settings [17–20].

The DOTS and Stop TB Strategy have been based on passive case detection at public health facilities and achieved remarkable results in scaling up quality-assured microscopy and TB case detection over the first 15 years of implementation during DOTS expansion [21]. However, in many countries as well as at a global level, these improvements have stalled, and renewed efforts and innovation are needed to continue towards the lofty goals in the End TB strategy and Stop TB Partnership’s Global Plan [22, 23]. This study shows that large increases in case detection can still be gained by better screening of facility attendees in a passive system as smaller studies have done [12]. However, almost 20% of notified SS+ TB cases in our study were identified through active outreach efforts among difficult to reach populations, highlighting the importance of moving outside the passive system to reach more people that may have TB but do not have proper access to services.

Findings from the three strategies had a number of other relevant outcomes. Consistent with other experiences of contact investigation [24, 25], we found that 1.6% of contacts had SS+ TB. However, there has been a great heterogeneity of results and interpretations from contact investigation studies, even within countries, depending on the setting [24, 26–27].

The similar NNS of those living in IDP camps and people seeking care in health facilities suggests major problems of access and poor health faced by IDPs, and they remained consistent over a two year period while yield from contact investigation showed a large drop in the second year. The likely reason for this drop in yield among household contacts is comprehensive coverage during year one which captured a majority of prevalent SS+ cases, while the second year saw increases in screening with less specificity. The lower NNS found for women in both years among both household contacts and IDPs is indicative of the more limited access to health care women encounter compared to men. It is not surprising that in residential settings, the higher NNS was found for women, as women are less likely to access care when travel is required and they are already remotely located. Focusing resources on key populations for TB, such as mobile groups with generally poor access to care, can identify many people with TB and greatly improve TB case notifications overall [17]. TB prevalence is estimated to be much higher among men than in women in many settings globally [1]. Yet in rural areas of Afghanistan, our findings seem to indicate that if outreach efforts prioritize case-finding among women, large numbers of active TB cases can be found and treated among women, exceeding those of men. Sabawoon and Soto examined Afghanistan’s SS+ PTB cases reported to WHO in 2007 and found that 31.5% came from men while 68.5% occurred among females, with respective incidence rates of 26.7 and 60.0 per 100,000 [28].

Our findings also support the idea that NNS cannot be interpreted in a uniform way across all settings and risk groups [29], and that the simplicity and cost of screening health facility attendees needs to be weighed against the yield and cost of outreach for key populations, the benefits of reaching people who have poor access to care, and the difficulty in measuring good (or poor) access to care [9].

Although systematic screening was conducted within the facilities, it is worth noting that we still likely missed many people with TB disease. The findings of recent TB prevalence surveys across Asia have shown that in many countries the majority of people with prevalent bacteriologically positive pulmonary TB may not report symptoms, and only a small fraction will be identified with smear microscopy [30]. While our approach clearly improved the numbers of people with TB treated and notified, many more could have been identified with better screening tools such as chest x-ray [31, 32]. Although more sensitive diagnostics such as Xpert MTB/RIF will identify more patients with bacteriologically positive TB, early studies around Xpert MTB/RIF implementation have shown no impact on overall TB treatment initiation due to empiric treatment [33–35]. Other situational factors during the second year may have also affected the project. Deteriorating security in the project area led to underutilization of some health facilities by the community, high staff turnover, and reduction in NTP quarterly TB review meetings which also occurred due to shortage of funds. Such issues are not avoidable in settings with high insecurity and minimal health system funding.

Despite the increases in notifications, treatment outcomes did not change between the baseline and intervention period, supporting findings that active case finding did not seem to improve patient outcomes [36]. With very high baseline treatment success rates, improving them significantly may be challenging.

Our study has a number of limitations. Because the study was not a strictly controlled trial, other factors may have influenced case notifications, and it should be noted that the active case finding yield exceeded official notifications in the intervention. This could be a result of people traveling into the intervention area to receive care through the project, but receiving treatment outside of the intervention area and/or pre-treatment loss to follow-up as documented in other studies [37]. People will travel to find the best and most convenient treatment options; therefore, ensuring that they are actually receiving treatment is vital.

We are unable to determine the impact of the individual interventions on additional notifications, similar to other studies looking at multiple case finding interventions implemented in the same place and time [38, 39]. Measuring and comparing the impact of specific strategies on additional notifications should be part of future research as some efforts may simply identify people with TB earlier, rather than in increased numbers. Based on the additional notifications in the analysis, many TB cases would not have been detected under routine conditions, or would have been diagnosed with a long delay. Many of the IDPs and household contacts with TB may not have been identified and were certainly identified earlier through the outreach, although we did not measure early case detection as some other studies have done [40,41]. What effect this kind of early and increased case detection has on transmission, prevalence and ultimately incidence is unclear. Other ACF studies [8,42] as well as modelling work [43,44] have suggested that ACF can reduce TB prevalence over a period of years, while others have not [11]. Also, a cost effectiveness analysis was unfortunately not possible given the scope of cost data we managed to collect, but is recognized as an important element of future ACF projects to enrich and contextualize results.

While security concerns continue to pose a challenge to TB control efforts in Afghanistan, our findings demonstrate that TB control programs can function effectively in unstable settings and targeted interventions can produce dramatic results even when notifications are stagnant or decreasing. However, funding remains a major obstacle to TB control efforts in Afghanistan as it is in many countries with high burdens of TB. Only 8% of TB control funding in Afghanistan was domestic at the time of our intervention, highlighting the reliance on international donors, primarily the Global Fund [45]. The Global Fund has placed improving case detection as the leading TB target for its current strategy [46]. While well-designed interventions can show improvements in case notification, the impact is diluted as the area under evaluation enlarges. For large improvements to be realized at a national level, these types of interventions must truly be scaled up. The lessons learned from this and other interventions to improve case detection should be taken into account when countries are planning developing National Strategic Plans, and concept notes for Global Fund and other donors. In order to scale up these interventions and show an impact as we move towards the goal of TB elimination, much more funding will be needed than previously thought, according to the Stop TB Partnership’s Global Plan to Stop TB 2016–2020 [23].

## Conclusion

These impressive gains build upon the paucity of published literature on TB activities in Afghanistan, yet the question that remains is how to sustain these improvements. Our findings should be taken into account when building on Afghanistan’s national TB strategy. Multi-faceted interventions that improve the identification of people for testing within health facilities, as well as targeted active outreach to key populations with poor access to care can increase the numbers of people treated for TB and save lives.

## Supporting Information

S1 Dataset Age and sex disaggregated data for three case finding approaches. (DOCX)

S2 Dataset Detailed case notification and treatment outcome data among 47 health facilities. (DOCX)
