# Supplementary material for: An Evaluation of Passive and Active Approaches to Improve Tuberculosis Notifications in Afghanistan
Source: PLoS One. 2016 Oct 4;11(10):e0163813. doi: 10.1371/journal.pone.0163813 (PMC5049786; doi:10.1371/journal.pone.0163813)
Supplement: S1 Dataset — (DOCX) [file pone.0163813.s001.docx]

| **Table S1A. Data for Case Finding Approach Among IDPs.** | | | | | |  |  |  |
| --- | --- | --- | --- | --- | --- | --- | --- | --- |
| **No** | **Process Indicators** | **Sex** | **Year 1** | **Year 2** | **Total** | \| **Table S1B. IDP New SS+ Data Disaggregated by Age and Sex During Years 1 and 2.** \| \| \| \| \| \| \| \| \| \| \| \| --- \| --- \| --- \| --- \| --- \| --- \| --- \| --- \| --- \| --- \| --- \| \|  \| **Sex** \| **0-4** \| **5 to 14** \| **15-24** \| **25-34** \| **35-44** \| **45-54** \| **55-64** \| **>65** \| **Total** \| \| Year 1 \| M \| 0 \| 3 \| 22 \| 18 \| 22 \| 28 \| 19 \| 22 \| **134** \| \| F \| 0 \| 8 \| 34 \| 41 \| 43 \| 44 \| 34 \| 20 \| **224** \| \| Year 2 \| M \| 0 \| 2 \| 14 \| 26 \| 9 \| 14 \| 19 \| 26 \| **110** \| \| F \| 0 \| 8 \| 39 \| 36 \| 25 \| 29 \| 29 \| 19 \| **185** \| \| **Total** \| M \| **0** \| **5** \| **36** \| **44** \| **31** \| **42** \| **38** \| **48** \| **244** \| \| F \| **0** \| **16** \| **73** \| **77** \| **68** \| **73** \| **63** \| **39** \| **409** \| | | |
| 1 | Number Screened | Men | 33829 | 32617 | **66446** |  |  |  |
|  |  | Women | 34590 | 33350 | **67940** |  |  |  |
|  |  | Children | 87478 | 84341 | **171819** |  |  |  |
|  |  | **Total** | **155897** | **150308** | **306205** |  |  |  |
| 2 | Number identified as TB suspects | Men | 2043 | 1498 | **3541** |  |  |  |
|  |  | Women | 3096 | 2268 | **5364** |  |  |  |
|  |  | **Total** | **5139** | **3766** | **8905** |  |  |  |
| 3 | Number TB suspects examined for TB | Men | 2043 | 1468 | **3511** |  |  |  |
|  |  | Women | 3096 | 2229 | **5325** |  |  |  |
|  |  | **Total** | **5139** | **3697** | **8836** |  |  |  |
| 4 | Number of identified new SS+ TB cases | Men | 134 | 110 | **244** |  |  |  |
|  |  | Women | 224 | 185 | **409** |  |  |  |
|  |  | **Total** | **358** | **295** | **653** |  |  |  |
| 5 | Number of identified all forms TB cases | Men | 146 | 129 | **275** |  |  |  |
|  |  | Women | 243 | 217 | **460** |  |  |  |
|  |  | **Total** | **389** | **346** | **735** |  |  |  |
| 6 | Number of new SS+ TB cases enrolled on treatment | Men | 134 | 110 | **244** |  |  |  |
|  |  | Women | 224 | 185 | **409** |  |  |  |
|  |  | **Total** | **358** | **295** | **653** |  |  |  |
| 7 | Number of all forms TB cases enrolled on treatment | Men | 146 | 129 | **275** |  |  |  |
|  |  | Women | 243 | 217 | **460** |  |  |  |
|  |  | **Total** | **389** | **346** | **735** |  |  |  |
| **Table S1C. Data for Case Finding Approach Among HHCs of SS+ TB Patients.** | | | | | | | |  |
| **No** | **Process Indicators** | **Sex** | **Year 1** | **Year 2** | | **Total** | |  |
| 1 | Number screened | Men | 3471 | 4697 | | 8168 | |  |
|  |  | Women | 3761 | 4716 | | 8477 | |  |
|  |  | **Total** | **7232** | **9413** | | **16645** | |  |
| 2 | Number identified as TB suspects | Men | 666 | 1335 | | 2001 | |  |
|  |  | Women | 814 | 1424 | | 2238 | |  |
|  |  | **Total** | **1480** | **2759** | | **4239** | |  |
| 3 | Number TB suspects examined for TB | Men | 666 | 1335 | | 2001 | |  |
|  |  | Women | 814 | 1424 | | 2238 | |  |
|  |  | **Total** | **1480** | **2759** | | **4239** | |  |
| 4 | Number of identified total SS+ TB cases | Men | 44 | 51 | | 95 | |  |
|  |  | Women | 92 | 81 | | 173 | |  |
|  |  | **Total** | **136** | **132** | | **268** | |  |
| 5 | Number of identified all forms TB cases | Men | 51 | 56 | | 107 | |  |
|  |  | Women | 107 | 90 | | 197 | |  |
|  |  | **Total** | **158** | **146** | | **304** | |  |
| 6 | Number of total SS+ TB cases enrolled on treatment | Men | 44 | 51 | | 95 | |  |
|  |  | Women | 92 | 81 | | 173 | |  |
|  |  | **Total** | **136** | **132** | | **268** | |  |
| 7 | Number of all forms TB cases enrolled on treatment | Men | 51 | 56 | | 107 | |  |
|  |  | Women | 107 | 90 | | 197 | |  |
|  |  | **Total** | **158** | **146** | | **304** | |  |

| **Table S1D. Data for Case Finding Approach Among 47 Health Facilities.** | | | | |
| --- | --- | --- | --- | --- |
| **No** | **Process Indicators** | **Year 1** | **Year 2** | **Total** |
| 1 | Total number screened | 889120 | 810157 | 1699277 |
| 2 | Number identified as TB suspects | 24001 | 25678 | 49679 |
| 3 | Number TB suspects examined for TB | 22228 | 24535 | 46763 |
| 4 | Number of identified total SS+ TB cases | 1986 | 2139 | 4125 |
| 5 | Number of identified all forms TB cases | 3540 | 4584 | 8124 |
| 6 | Number of total SS+ TB cases enrolled on treatment | 1986 | 2139 | 4125 |
| 7 | Number of all forms TB cases enrolled on treatment | 3540 | 4584 | 8124 |

| **Table S1E. 47 Health Facilities New SS+ Data Disaggregated by Age and Sex During Years 1 and 2.** | | | | | | | | | | |
| --- | --- | --- | --- | --- | --- | --- | --- | --- | --- | --- |
|  | **Sex** | **0-4** | **05 to 14** | **15-24** | **25-34** | **35-44** | **45-54** | **55-64** | **>65** | **Total** |
| Year 1 | M | 0 | 34 | 111 | 108 | 74 | 94 | 101 | 83 | **605** |
|  | F | 0 | 62 | 229 | 311 | 233 | 174 | 127 | 87 | **1223** |
| Year 2 | M | 0 | 38 | 147 | 124 | 92 | 84 | 98 | 94 | **677** |
|  | F | 0 | 47 | 256 | 318 | 239 | 177 | 162 | 95 | **1294** |
| **Total** | M | **0** | **72** | **258** | **232** | **166** | **178** | **199** | **177** | **1282** |
|  | F | **0** | **109** | **485** | **629** | **472** | **351** | **289** | **182** | **2517** |

| **Table S1F. Combined Data For 3 Case Finding Approaches.** | | | | |
| --- | --- | --- | --- | --- |
| **No** | **Process Indicators** | **Year 1** | **Year 2** | **Total** |
| 1 | Total number screened | 1052249 | 969878 | 2022127 |
| 2 | Number Identified as TB Suspects | 30620 | 32203 | 62823 |
| 3 | Number TB suspects examined for TB | 28847 | 30991 | 59838 |
| 4 | Number identified as SS+ TB cases (Total SS+) | 2480 | 2566 | 5046 |
| 5 | Number identified as TB All Forms | 4087 | 5076 | 9163 |
| 6 | Number SS+ enrolled on treatment | 2480 | 2566 | 5046 |
| 7 | Number TB All forms enrolled on treatment | 4087 | 5076 | 9163 |

| **Table S1G. 3 Case Finding Approaches New SS+ Data Disaggregated by Age and Sex During Years 1 and 2.** | | | | | | | | | | |
| --- | --- | --- | --- | --- | --- | --- | --- | --- | --- | --- |
|  | **Sex** | **0-4** | **5 – 14** | **15–24** | **25–34** | **35–44** | **45–54** | **55–64** | **> 65** | **Total** |
| Year 1 | M | 0 | 36 | 131 | 126 | 96 | 121 | 120 | 105 | **735** |
|  | F | 0 | 70 | 260 | 351 | 274 | 218 | 161 | 107 | **1441** |
| Year 2 | M | 0 | 37 | 160 | 150 | 102 | 104 | 118 | 120 | **791** |
|  | F | 0 | 59 | 287 | 347 | 264 | 199 | 190 | 115 | **1461** |
| **Total** | M | **0** | **73** | **291** | **276** | **198** | **225** | **238** | **225** | **1526** |
|  | F | **0** | **129** | **547** | **698** | **538** | **417** | **351** | **222** | **2902** |
