# Supplementary material for: An Evaluation of Passive and Active Approaches to Improve Tuberculosis Notifications in Afghanistan
Source: PLoS One. 2016 Oct 4;11(10):e0163813. doi: 10.1371/journal.pone.0163813 (PMC5049786; doi:10.1371/journal.pone.0163813)
Supplement: S2 Dataset — (DOCX) [file pone.0163813.s002.docx]

| **Table S2A. 47 Health Facilities Case Notification Data Q4-2007 to Q4-2012.** | | | | | | | | | |
| --- | --- | --- | --- | --- | --- | --- | --- | --- | --- |
| **No** | **Historical Quarterly Reporting Period** | **Pulmonary bacteriologically positive or sputum smear microscopy positive** | | | **New SS-** | **New SS Not Done** | **New EP** | **Others** | **Total Cases** |
|  |  |  |  |  |  |  |  |  |  |
|  |  | **New cases** | **Previously Treated** | **Total SS+** |  |  |  |  |  |
| 1 | Q4 2007 | 312 | 21 | **333** | 280 | NA* | 116 | NA | **729** |
| 2 | Q1 2008 | 353 | 44 | **397** | 298 | NA | 159 | NA | **854** |
| 3 | Q2 2008 | 388 | 37 | **425** | 479 | NA | 199 | NA | **1103** |
| 4 | Q3 2008 | 325 | 31 | **356** | 452 | NA | 159 | NA | **967** |
| 5 | Q4 2008 | 286 | 23 | **309** | 268 | NA | 146 | NA | **723** |
| 6 | Q1 2009 | 375 | 34 | **409** | 303 | NA | 212 | NA | **924** |
| 7 | Q2 2009 | 328 | 19 | **347** | 275 | NA | 151 | NA | **773** |
| 8 | Q3 2009 | 280 | 18 | **298** | 205 | NA | 135 | NA | **638** |
| 9 | Q4 2009 | 307 | 17 | **324** | 225 | NA | 150 | NA | **699** |
| 10 | Q1 2010 | 302 | 21 | **323** | 168 | 116 | 151 | 7 | **765** |
| 11 | Q2 2010 | 352 | 26 | **378** | 128 | 246 | 176 | 32 | **960** |
| 12 | Q3 2010 | 318 | 35 | **353** | 119 | 308 | 196 | 12 | **988** |
| 13 | Q4 2010 | 350 | 40 | **390** | 114 | 141 | 156 | 47 | **848** |
| 14 | Q1 2011 | 618 | 62 | **680** | 171 | 90 | 158 | 51 | **1150** |
| 15 | Q2 2011 | 659 | 58 | **717** | 125 | 84 | 188 | 41 | **1155** |
| 16 | Q3 2011 | 549 | 46 | **595** | 80 | 106 | 127 | 26 | **934** |
| 17 | Q4 2011 | 441 | 45 | **486** | 72 | 110 | 133 | 40 | **841** |
| 18 | Q1 2012 | 460 | 47 | **507** | 114 | 84 | 156 | 47 | **908** |
| 19 | Q2 2012 | 494 | 44 | **538** | 186 | 231 | 229 | 17 | **1201** |
| 20 | Q3 2012 | 446 | 29 | **475** | 155 | 210 | 185 | 65 | **1090** |
| 21 | Q4 2012 | 411 | 43 | **454** | 126 | 184 | 188 | 84 | **1036** |

| **Table S2B. 47 Health Facilities Treatment Outcome Data for New SS+ TB Patients.** | | | | | | | | | |  |
| --- | --- | --- | --- | --- | --- | --- | --- | --- | --- | --- |
| **No** | **Year** | **Total Number of Cases Registered for Treatment** | **Treatment Outcomes** | | | | | | | Total |
|  |  |  | **Cured** | **Treatment Completed** | **Default** | **Treatment Failure** | **Death** | **Transfer out** | **Not Evaluated** |  |
| 1 | Year 1 | 2110 | 1834 | 72 | 20 | 5 | 26 | 103 | 50 | **2110** |
| 2 | Year 2 | 2252 | 1939 | 62 | 25 | 6 | 24 | 151 | 45 | **2252** |
